# Positron emission tomography imaging of tumor angiogenesis and monitoring of antiangiogenic efficacy using the novel tetrameric peptide probe ^64^Cu-cyclam-RAFT-c(-RGDfK-)_4_

**DOI:** 10.1007/s10456-012-9281-1

**Published:** 2012-05-29

**Authors:** Zhao-Hui Jin, Takako Furukawa, Michael Claron, Didier Boturyn, Jean-Luc Coll, Toshimitsu Fukumura, Yasuhisa Fujibayashi, Pascal Dumy, Tsuneo Saga

**Affiliations:** 1 Molecular Imaging Center, National Institute of Radiological Sciences, Anagawa 4-9-1, Inage-ku, Chiba 263-8555 Japan; 2 Département de Chimie Moléculaire, UMR5250, CNRS, Université Joseph Fourier, Grenoble, France; 3 INSERM U823, Institut Albert Bonniot, Université Joseph Fourier, Grenoble, France

**Keywords:** Angiogenesis, Positron emission tomography, α_V_β_3_ Integrin, Multimeric RGD peptide, TSU-68

## Abstract

^64^Cu-cyclam-RAFT-c(-RGDfK-)_4_ is a novel multimeric positron emission tomography (PET) probe for α_V_β_3_ integrin imaging. Its uptake and α_V_β_3_ expression in tumors showed a linear correlation. Since α_V_β_3_ integrin is strongly expressed on activated endothelial cells during angiogenesis, we aimed to determine whether ^64^Cu-cyclam-RAFT-c(-RGDfK-)_4_ PET can be used to image tumor angiogenesis and monitor the antiangiogenic effect of a novel multi-targeted tyrosine kinase inhibitor, TSU-68. Athymic nude mice bearing human hepatocellular carcinoma HuH-7 xenografts, which expressed negligible α_V_β_3_ levels on the tumor cells, received intraperitoneal injections of TSU-68 or the vehicle for 14 days. Antiangiogenic effects were determined at the end of therapy in terms of ^64^Cu-cyclam-RAFT-c(-RGDfK-)_4_ uptake evaluated using PET, biodistribution assay, and autoradiography, and they were compared with microvessel density (MVD) determined by CD31 immunostaining. ^64^Cu-cyclam-RAFT-c(-RGDfK-)_4_ PET enabled clear tumor visualization by targeting the vasculature, and the biodistribution assay indicated high tumor-to-blood and tumor-to-muscle ratios of 31.6 ± 6.3 and 6.7 ± 1.1, respectively, 3 h after probe injection. TSU-68 significantly slowed tumor growth and reduced MVD; these findings were consistent with a significant reduction in the tumor ^64^Cu-cyclam-RAFT-c(-RGDfK-)_4_ uptake. Moreover, a linear correlation was observed between tumor MVD and the corresponding standardized uptake value (SUV) (r = 0.829, *P* = 0.011 for SUV_mean_; r = 0.776, *P* = 0.024 for SUV_max_) determined by quantitative PET. Autoradiography and immunostaining showed that the distribution of intratumoral radioactivity and tumor vasculature corresponded. We concluded that ^64^Cu-cyclam-RAFT-c(-RGDfK-)_4_ PET can be used for in vivo angiogenesis imaging and monitoring of tumor response to antiangiogenic therapy.

**Electronic supplementary material:**

The online version of this article (doi:10.1007/s10456-012-9281-1) contains supplementary material, which is available to authorized users.

## Introduction

Angiogenesis, the process by which new blood vessels are formed from the existing vasculature, is an important target and indicator in the therapy and prognosis of cancer, because it is a key feature of malignant solid tumors and plays a critical role in tumor growth, invasion, and metastasis [1]. This process comprises several steps and is jointly regulated by the nature of the tumor and a complex network of several cell types and various growth factors/receptors and signaling pathways involved in endothelial proliferation, migration, and tube formation [2, 3]. Several different angiogenesis inhibitors have been developed for blocking different steps of angiogenesis, whereby the formation of new blood vessels can be suppressed and the growth or spread of tumors can be stopped or slowed [4–6]. Several agents have even been approved for therapeutic use in cancer patients and many are undergoing clinical trials. However, despite the rapid progress in drug development, the choice of an appropriate assay to evaluate the treatment response remains a challenge. Unlike cytotoxic chemotherapeutic drugs, angiogenesis inhibitors are typically cytostatic, that is, they slow or stop tumor growth rather than causing tumor shrinkage. Therefore, the routine methods for evaluating chemotherapeutic efficiency, including parameters such as changes in tumor volume or morphology detected by computed tomography (CT) and magnetic resonance imaging (MRI), may not be suitable for assessing the response to antiangiogenic treatment [7]. Dynamic contrast-enhanced CT and MRI can be used to evaluate tumor blood flow, blood volume, and vascular permeability, but they cannot effectively quantify changes in tumor vascularity [7]. The impact of molecular imaging on drug evaluation and development has been well reviewed [8]. Positron emission tomography (PET) with the tracer fluorine-18(^18^F)-labeled fluorodeoxyglucose (FDG), or ^18^F-FDG PET, has been widely used in clinical oncology for diagnosis, staging, and monitoring of treatment effects. This technique is based on the preferential uptake of the tracer by tumors having a high glucose metabolic activity [9]. However, some studies that used ^18^F-FDG PET to monitor antiangiogenic efficiency showed no significant change in tumor tracer uptake [10, 11].

α_V_β_3_ Integrin, which is strongly expressed on activated endothelial cells during angiogenesis, is one of the most extensively studied members of the integrin family, which comprises heterodimeric transmembrane glycoprotein receptors composed of 2 noncovalently associated α and β subunits [12]. By specifically binding to extracellular matrix proteins via the tripeptide sequence Arg-Gly-Asp (RGD), α_V_β_3_ integrin plays an important role in endothelial cell survival and migration [12]. Pentapeptides containing cyclic RGD (cRGDs) are optimized synthetic ligands that have a high affinity and selectivity for α_V_β_3_ integrin [13], and a series of radiolabeled cRGD-containing peptides have been introduced for the noninvasive imaging of α_V_β_3_ integrin expression [14, 15]. However, since tumor vessels represent about 5 % of the volume of a tumor and α_V_β_3_ integrin is expressed only on activated endothelial cells and not on quiescent endothelial cells, evaluation of angiogenesis by targeting α_V_β_3_ integrin is expected to be quite challenging; it would require a tracer with high specificity, strong affinity, and favorable pharmacokinetics [16, 17]. Few reports have shown that an RGD-based PET probe can be effectively used to monitor the chemotherapeutic [18, 19] or antiangiogenic treatment response [10, 11, 20, 21]. We recently developed a multimeric cRGD-containing RAFT-c(-RGDfK-)_4_-based PET probe, known as ^64^Cu-cyclam-RAFT-c(-RGDfK-)_4_. It was synthesized by separately grafting 4 cyclo(-RGDfK-) peptide monomers onto the upper side of the regioselectively addressable functionalized template (RAFT) cyclic decapeptide platform [22]. Further, it was labeled with ^64^Cu via the chelating agent cyclam conjugated to RAFT on the lower side [23, 24]. In the present study, we evaluated the potential of this probe to enable the visualization and quantification of tumor angiogenesis and monitoring of the tumor response to the novel angiogenesis inhibitor TSU-68, a small-molecule oxindole compound.

## Materials and methods

### Generation of cyclam-RAFT-c(-RGDfK-)_4_ and radiolabeling with ^64^Cu

Cyclam-RAFT-c(-RGDfK-)_4_ (molecular weight: 4,119.6) was designed and synthesized as reported previously [23]. The peptide was radiolabeled with ^64^Cu in accordance with our previous report with minor modifications [24]. In brief, the peptide was dissolved in 10 % HEPES/90 % MeOH (HEPES, 10 mM, pH 7.0) just before radiolabeling or in dimethyl sulfoxide (DMSO) (the aliquots can be kept at −20 °C for long-term storage). ^64^CuCl_2_ powder was reconstituted in ammonium citrate buffer (100 mM, pH 5.5). The peptide (0.4 mM) and ^64^CuCl_2_ (1.18 MBq/μl) were mixed in a 1/1 (vol/vol) ratio and incubated in a 37 °C water bath for 60 min. The radiolabeling efficiency was determined via reversed phase high-performance liquid chromatography (RP-HPLC) or thin-layer chromatography (TLC) with autoradiography using a bioimaging analyzer according to a previously described method [24]. It was found to be >99 % for ^64^Cu-labeled cyclam-RAFT-c(-RGDfK-)_4_, and the specific radioactivity was about 3 MBq/nmol. The maximum specific radioactivity that could be achieved for this peptide was ~37 MBq/nmol (unpublished data).

### Cell lines and animal tumor models

Human hepatocellular carcinoma HuH-7 cells were purchased from the JCRB Cell Bank (Osaka, Japan) and cultured in RPMI 1640 medium (Wako Pure Chemical Industries, Ltd., Osaka, Japan) supplemented with 10 % fetal bovine serum (Nichirei Biosciences, Inc., Tokyo, Japan), 50 U/ml penicillin, and 50 μg/ml streptomycin (Wako Pure Chemical Industries, Ltd.). α_V_β_3_-Negative HEK293(β_1_) and α_V_β_3_-overexpressing HEK293(β_3_) cells (kindly provided by J.-F. Gourvest, Aventis, France), stable transfectants of the human embryonic kidney HEK293 cell line with human integrin β_1_ and β_3_ subunits, respectively, were cultured under conditions described previously [25]. All the cells were cultured at 37 °C in a humidified 95  % air/5  % CO_2_ atmosphere.

Animal procedures were approved by the Institutional Animal Care and Use Committee of the National Institute of Radiological Sciences. Cell suspensions containing 2 × 10^6^ HuH-7, 1 × 10^7 ^HEK293(β_1_), or 2 × 10^7 ^HEK293(β_3_) cells were implanted by subcutaneous (s.c.) injection with a 25-gauge needle into the flank of female athymic nude mice (BALB/cAJcl-*nu/nu*; CLEA Japan, Inc., Tokyo, Japan). The suspensions also contained 50 vol % Matrigel (BD Biosciences, Bedford, MA) to facilitate tumor development. Mice with tumors of diameter approximately 5–10 mm were selected for subsequent experiments.

### Flow cytometric analysis

The expression of α_V_β_3_ integrin on the surface of HuH-7 cells was analyzed by labeling the cells with R-phycoerythrin-conjugated anti-human α_V_β_3_ monoclonal antibody (clone LM609; Millipore Corporation, Temecula, CA), which was detected using the Guava EasyCytePlus Flow Cytometry System (Millipore Corporation) according to a previously described method [24]. α_V_β_3_-Negative HEK293(β_1_) and α_V_β_3_-overexpressing HEK293(β_3_) cells were simultaneously analyzed as the negative and positive controls, respectively.

### Immunohistochemical and histological analyses

Frozen tumor sections (7-μm thick) were fixed in acetone, stained with mouse anti-human α_V_β_3_ monoclonal antibody (LM609; 1:100 dilution; Chemicon, Temecula, CA), and examined using the M.O.M. Immunodetection Kit (Vector Laboratories, Inc., Burlingame, CA). Purified rat anti-mouse CD31 monoclonal antibody (1:1,500 dilution; BD Biosciences) bound to biotinylated rabbit anti-rat secondary antibody (1:600 dilution; Dako, Glostrup, Denmark) was used for microvessel staining, and the Histostain-*plus* Bulk Immunostaining Kit (LAB-SA Detection System; Invitrogen, Camarillo, CA) was used for detection, with diaminobenzidine (DAB) as the chromogen. Nuclei were counter-stained with hematoxylin. Serial sections of the tumors were stained with hematoxylin and eosin (HE) for histological examination.

### Antiangiogenesis therapy

TSU-68 (synonym: SU6668; molecular weight: 310.35) was purchased from Selleck Chemicals (Houston, TX). It was dissolved in DMSO at 30 mg/ml, and aliquots were stored at −20 °C until use. For each set of experiments, mice bearing HuH-7 tumors were divided into 2 groups (n = 4–6). The treatment group received intraperitoneal (i.p.) injections of TSU-68 (75 mg kg^−1^ d^−1^ in 50 μl of DMSO) for 14 days (days 1–14), and the control animals received i.p. injections of the vehicle alone (50 μl of DMSO). Every 2 days throughout the experiment, the body weight (g) was recorded, and the tumors were measured simultaneously by using a vernier caliper. Tumor volume (mm^3^) was determined using the formula 0.5 × length × width^2^, and the fold change in volume was calculated by dividing the obtained value by the tumor volume on the day before the treatment was started (day 0). The therapeutic response to TSU-68 was assessed on the day after the final drug injection (day 15).

### Immunofluorescence staining and MVD measurement

Frozen tumor sections (7- or 30-μm thick) were fixed in acetone, stained with purified rat anti-mouse CD31 monoclonal antibody, and visualized using Alexa Fluor 488-conjugated goat anti-rat antibody (1:200 dilution; Invitrogen). Unlike the pan-endothelial cell marker CD31, CD105 is reported to be specifically an activated endothelial cell marker [26]. To compare the pattern of staining between them, serial sections (7-μm thick) were stained with the CD31 antibody or rat anti-mouse CD105 monoclonal antibody (1:500 dilution; BD Biosciences) and visualized using Alexa Fluor 488-conjugated goat anti-rat secondary antibody. Double staining was conducted for CD31 and CD61, the mouse β_3_ integrin subunit, wherein 7-μm-thick sections were simultaneously treated with the CD31 antibody and purified Armenian hamster anti-mouse CD61 antibody (1:50 dilution; BD Biosciences) and then coincubated with Alexa Fluor 594-conjugated goat anti-rat secondary antibody (1:200 dilution; Invitrogen) and Alexa Fluor 488-conjugated goat anti-Armenian hamster secondary antibody (1:100 dilution; Jackson Immunoresearch, West Grove, PA). The slides were then mounted with mounting agent (Dapi-Fluoromount-G™; SouthernBiotech, Birmingham, AL) containing 4′,6-diamidino-2-phenylindole (DAPI) for nucleus staining. Fluorescence images were acquired with an epifluorescence microscope (Olympus X61) equipped with an image tiling system (e-Tiling; Mitani Corporation, Fukui, Japan), which enabled the creation of a high-resolution image depicting whole tumor sections from separately captured photos.

The interwoven microvessel network observed in the HuH-7 tumor sections made it difficult to count the number of CD31-positive vessels. Therefore, the percentage of the CD31-stained area versus the area of the entire section, as assessed using the WinROOF image analysis software (version 6.5; Mitani Corporation), was used to express the relative microvessel density (MVD) (%) of the tumor.

### Biodistribution study

HuH-7 tumor-bearing mice received tail vein (intravenous; i.v.) injections of 0.74 MBq ^64^Cu-cyclam-RAFT-c(-RGDfK-)_4_, and at the indicated times of 1 and/or 3 h postinjection (p.i.), the mice were sacrificed, and the tumor and normal organs of interest were collected, weighed, and processed for radioactivity counting using a γ-counter with decay correction. The radioactivity uptake in the tumor and normal organs was expressed as a percentage of the injected dose per gram of tissue (% ID/g) normalized to a mouse body weight of 20 g.

### PET imaging

For PET imaging, the mice were i.v. injected with 11.1 MBq ^64^Cu-cyclam-RAFT-c(-RGDfK-)_4_, and at the indicated times (1 and/or 3 h p.i.,), static scans were acquired for 30 min using a small-animal PET system (Inveon; Siemens Medical Solutions USA, Inc., Malvern, PA) with the animals under 1 % isoflurane anesthesia. The acquired three-dimensional emission data were reconstructed using a maximum *a posterior* (MAP) reconstruction method with attenuation correction. Image display and analysis were performed using the ASIPro VM Micro PET Analysis software (Siemens Medical Solutions USA, Inc.). The radioactivity uptake in the tumor, expressed as standardized uptake value (SUV; corrected for body weight and injected radioactivity), was determined by selecting regions of interest (ROIs) encompassing the tumor on each of transverse frames and then using the 3D (VOI) dimensionality tool to link all the drawn ROIs to form a volume of interest. The maximum, mean, and minimum SUV (SUV_max_, SUV_mean_, and SUV_min_, respectively) were then acquired automatically.

### Autoradiographic examination

After PET imaging, the mice were sacrificed, and the tumors and kidneys were excised, embedded in Tissue-Tek OCT compound (Sakura Finetek, Torrance, CA), and frozen by immersion in *n*-hexane pre-cooled at −80°. Next, 30-μm-thick tumor sections or 5-μm-thick kidney sections were air-dried and exposed to an imaging plate (BAS-MS 2040; Fujifilm Co. Ltd., Tokyo, Japan), and after overnight exposure at −80°, the plate was scanned using a bioimaging analyzer (FLA-7000; Fujifilm Co. Ltd.). The sections were then stored at −80 °C until the radioactivity decayed to negligible levels, after which immunofluorescence staining was conducted.

### Statistical analysis

Quantitative data were given as mean ± standard deviation (SD). Statistical comparison between groups was performed using the unpaired *t* test, and *P* < 0.05 was considered significant. Correlation analysis was performed using Kaleida Graph (Synergy Software, Reading, PA).

## Results

### Use of HuH-7 xenograft as a tumor angiogenesis model

**Fig. 1:**
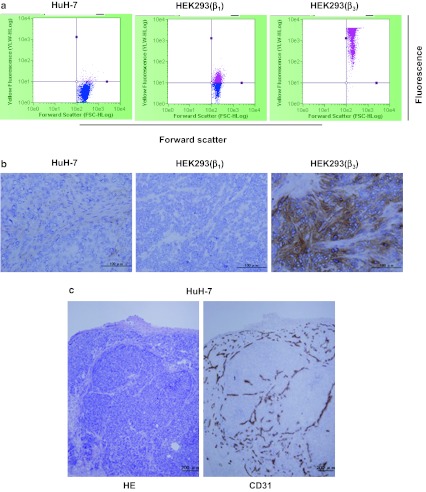
**a** Flow cytometric analysis for detecting α_V_β_3_ integrin expression on human hepatocellular carcinoma HuH-7 cells in culture. HEK293(β_1_) and HEK293(β_3_) were used as the negative and positive controls, respectively. **b** Immunohistochemical staining of human α_V_β_3_ integrin in HuH-7, HEK293(β_1_), and HEK293(β_3_) tumor xenografts. **c** HE staining and CD31 immunohistochemical staining of tumor vasculature in HuH-7 tumor xenografts

Human hepatocellular carcinoma HuH-7 cells, either in cell culture (Fig. 1a) or in the mouse xenografts (Fig. 1b), were found to express negligible levels of α_V_β_3_ integrin as compared to α_V_β_3_-positive HEK293(β_3_) and α_V_β_3_-negative HEK293(β_1_) cells or tumor xenografts, which were the positive and negative controls, respectively. HE and immunohistochemical staining of the endothelial cell marker CD31 showed that the HuH-7 tumor xenografts were highly vascularized, and nests of tumor cells were found surrounded by abundant microvessels (Fig. 1c).

### Biodistribution of ^64^Cu-cyclam-RAFT-c(-RGDfK-)_4_ and PET imaging in the HuH-7 tumor model

**Fig. 2:**
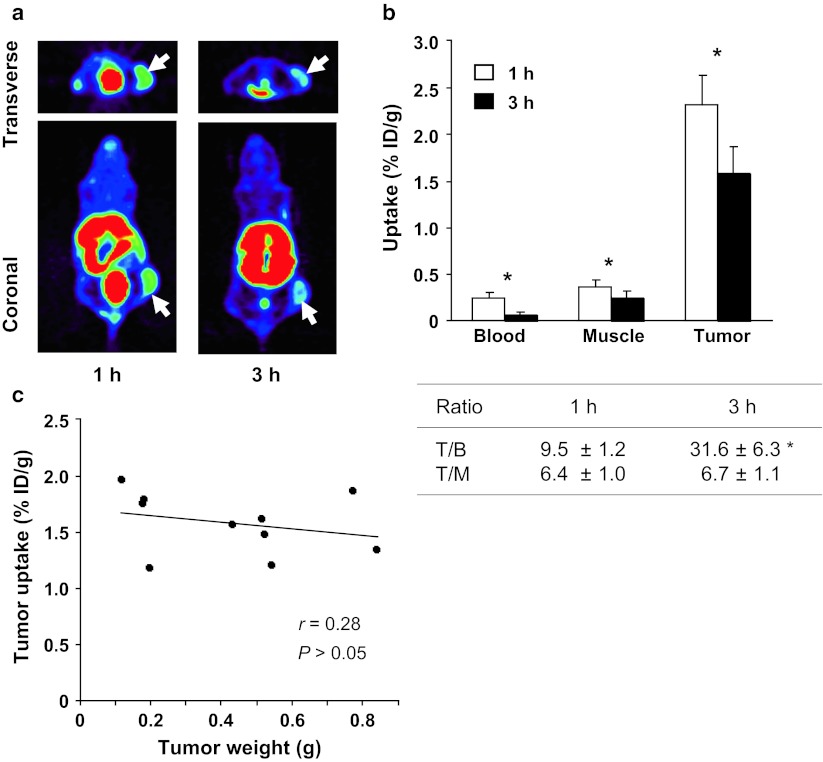
**a** Representative decay-corrected transverse and coronal PET images of nude mice bearing s.c. HuH-7 tumors at 1 and 3 h after i.v. injection of ^64^Cu-cyclam-RAFT-c(-RGDfK-)_4_ (11.1 MBq). The *arrows* indicate the tumor location. **b** Uptake of ^64^Cu-cyclam-RAFT-c(-RGDfK-)_4_ (0.74 MBq) in the tumor, blood, and muscle of HuH-7 tumor-bearing mice at 1 and 3 h after tracer injection (n = 4 and 10, respectively). Data are shown as mean (% ID/g) ± SD. *T/B* tumor-to-blood, *T/M* tumor-to-muscle. *P < 0.05 (1 h vs. 3 h). **c** Correlation analysis between tumor ^64^Cu-cyclam-RAFT-c(-RGDfK-)_4_ uptake and tumor weight (g). The uptake data are those corresponding to the 3 h time point in **b**

Figure 2a shows representative decay-corrected transverse and coronal PET images of HuH-7 tumor-bearing mice. The HuH-7 tumor could clearly be visualized at 1 and 3 h after injection of ^64^Cu-cyclam-RAFT-c(-RGDfK-)_4_, with higher radioactivity accumulation at 1 h than 3 h p.i. High kidney and urinary bladder uptake were also observed, indicating the rapid renal excretion of the agent. The biodistribution data of ^64^Cu-cyclam-RAFT-c(-RGDfK-)_4_ in the HuH-7 tumor-bearing mice at 1 and 3 h p.i. are shown in Fig. 2b. The tumor uptake was 2.32 ± 0.31 % ID/g and 1.59 ± 0.28 % ID/g at 1 and 3 h, respectively. The tumor-to-blood ratio was 9.5 ± 1.2 at 1 h and it markedly increased to 31.6 ± 6.3 at 3 h (*P* < 0.05), while the tumor-to-muscle ratio was 6.4 ± 1.0 and 6.7 ± 1.1 at 1 and 3 h p.i., respectively. The analysis of the correlation between tumor weight and tumor uptake of ^64^Cu-cyclam-RAFT-c(-RGDfK-)_4_ is presented in Fig. 2c. Neither a positive nor a negative correlation was found between these parameters, and this finding may indicate that the tumor size itself is not a critical factor influencing tracer uptake.

### Treatment effect of TSU-68 as assessed by tumor growth and MVD

**Fig. 3:**
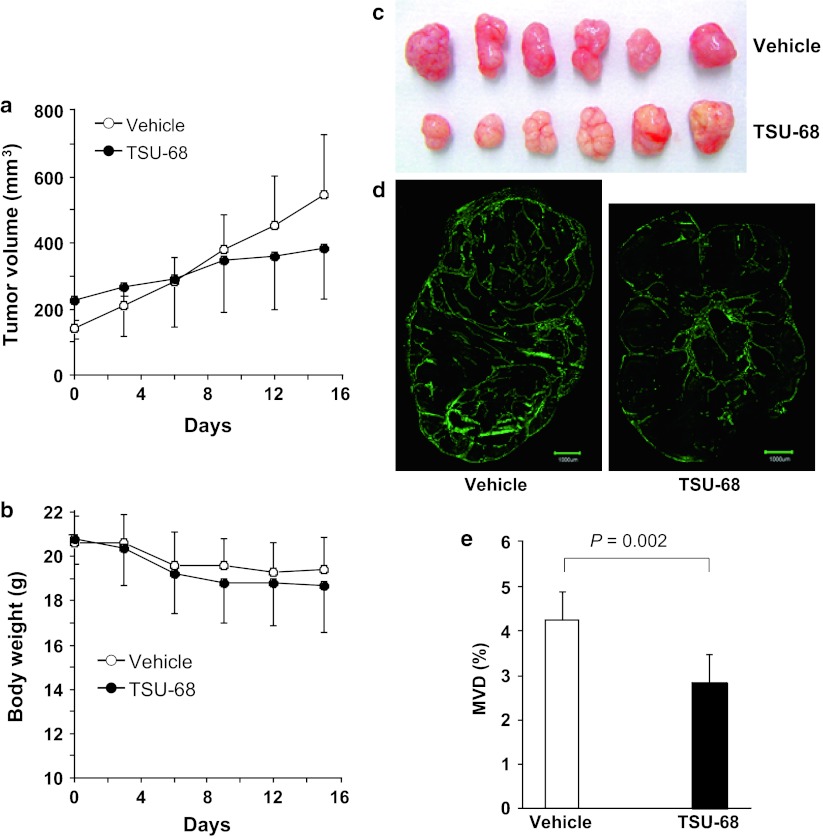
**a** Tumor growth curve of s.c. HuH-7 tumor-bearing mice treated with daily i.p. injections of TSU-68 (75 mg kg^−1^ d^−1^ in 50 μl of DMSO) or the vehicle alone (50 μl of DMSO). Data are presented as the mean ± SD of tumor volumes. Day 0: the day before treatment; days 1–14: treatment days; day 15: 1 day after treatment. **b** Changes in the body weight of TSU-68- and vehicle-treated mice. **c** Image of TSU-68- and vehicle-treated tumors excised on day 15. **d** Representative immunofluorescence staining of tumor sections from TSU-68- and vehicle-treated tumors using anti-mouse CD31 antibody (*green*). *Scale bar* = 1,000 μm. **e** The tumor MVD presented as the percentage of the CD31-positive area was compared between tumors from TSU-68- and vehicle-treated mice. All data presented in **a**–**e** are from the same set of experimental groups (n = 6 for each group)

HuH-7 tumor growth was compared between TSU-68- and DMSO only-treated mice (n = 6 for each group) (Fig. 3a). No significant difference was found in the initial tumor volumes between the groups. Daily administration of TSU-68 for 14 days tended to slow the tumor growth: on day 15, the tumor volume was 384 ± 148 mm^3^ for the treatment group versus 545 ± 178 mm^3^ for the control group (*P* > 0.05), corresponding to a tumor weight of 0.32 ± 0.14 g for the treatment group versus 0.45 ± 0.23 g for the control group (*P* > 0.05). Although direct comparison of tumor size showed no statistically significant differences, the fold change in tumor volume (days 3–15) from the baseline value (day 0) was found to be statistically significant (*P* < 0.05): 1.18 ± 0.19, 1.31 ± 0.22, 1.57 ± 0.32, 1.65 ± 0.47, and 1.79 ± 0.47 on days 3, 6, 9, 12, and 15, respectively, for the treatment group versus 1.48 ± 0.25, 2.0 ± 0.43, 2.67 ± 0.65, 3.17 ± 0.84, and 3.79 ± 1.01 on days 3, 6, 9, 12, and 15, respectively, for the control group. TSU-68 treatment seemed not to cause obvious side effects because no significant difference was observed in body weight between the TSU-68-treated and control mice (Fig. 3b), and no other signs of toxicity were noted. Photographs of the excised tumors on day 15 (Fig. 3c) showed that the tumors in the TSU-68-treated mice were paler than those in the control mice, indicating that the tumor vasculature was negatively affected by TSU-68 treatment. Comparison of CD31 immunofluorescence staining between whole-tumor sections from TSU-68-treated mice and control mice also revealed the same (Fig. 3d). Further, as seen in Fig. 3e, the MVD was significantly lower in the TSU-68-treated tumors than in the control tumors (2.84 ± 0.61 % vs. 4.26 ± 0.60 %; *P* = 0.002).

### Effect of TSU-68 on ^64^Cu-cyclam-RAFT-c(-RGDfK-)_4_ biodistribution in HuH-7 tumor-bearing mice

The biodistribution data of ^64^Cu-cyclam-RAFT-c(-RGDfK-)_4_ at 3 h p.i. in the TSU-68 and vehicle-treated HuH-7 tumor-bearing mice are presented in Table 1. Daily administration of TSU-68 for 14 days significantly reduced the tumor uptake of ^64^Cu-cyclam-RAFT-c(-RGDfK-)_4_ by 36 %. It also caused a significant reduction in tracer uptake in most organs examined, except the pancreas. Table 2 shows the results of another biodistribution assay performed after only 1 day of therapy (day 2), when the tumor volume change was still not significantly different between the groups. Before drug administration, the tumor volume was 218 ± 96 mm^3^ for the TSU-68 group versus 220 ± 96 mm^3^ for the control group (*P* > 0.05), and after 1 day of therapy, no significant difference was found either in the tumor volumes (222 ± 122 mm^3^ and 236 ± 113 mm^3^ for the TSU-68 and control groups, respectively) or in the fold changes in tumor volume (0.99 ± 0.16 and 1.06 ± 0.15 for the TSU-68 and control groups, respectively) between the groups. TSU-68 injection on 1 day tended to result in a reduced tumor uptake of ^64^Cu-cyclam-RAFT-c(-RGDfK-)_4_ as compared to the vehicle control (*P* = 0.085), which was consistent with a similar tendency noted in the corresponding tumor MVD. Tracer accumulation in examined major organs was not found to significantly differ between the groups. Further, a positive and significant correlation was observed between tumor tracer uptake and the corresponding MVD (correlation coefficient r = 0.711, *P* = 0.001) (Supplemental Fig. 1).

**Table 1:** Biodistribution of ^64^Cu-cyclam-RAFT-c(-RGDfK-)_4_ in HuH-7 tumor-bearing mice treated with TSU-68 or vehicle after 14 days of therapy

Organ	Vehicle (n = 5)	TSU-68 (n = 5)
Blood	0.07 ± 0.01	0.05 ± 0.01^a^
Heart	0.59 ± 0.08	0.37 ± 0.06^a^
Lung	1.58 ± 0.24	1.08 ± 0.21^a^
Liver	5.37 ± 0.36	3.75 ± 0.46^a^
Pancreas	0.61 ± 0.13	0.55 ± 0.15
Spleen	2.86 ± 0.49	1.65 ± 0.21^a^
Stomach	1.94 ± 0.34	1.40 ± 0.19^a^
Intestine	1.58 ± 0.14	0.96 ± 0.15^a^
Kidney	25.4 ± 3.48	16.5 ± 3.36^a^
Skin	2.21 ± 0.22	1.51 ± 0.28^a^
Muscle	0.31 ± 0.07	0.21 ± 0.03^a^
Bone	0.81 ± 0.23	0.47 ± 0.06^a^
Tumor	2.44 ± 0.93	1.57 ± 0.53^a^

Tissue radioactivity was assessed 3 h after injection of 0.74 MBq of the tracer and is expressed as % ID/g (mean ± SD)

^a^ *P* < 0.05 versus the vehicle-treated group

**Table 2:** Biodistribution of ^64^Cu-cyclam-RAFT-c(-RGDfK-)_4_ in HuH-7 tumor-bearing mice treated with TSU-68 or vehicle after 1 day of therapy

Organ	Vehicle (n = 9)	TSU-68 (n = 9)
Blood	0.11 ± 0.03	0.09 ± 0.02^ns^
Liver	4.97 ± 0.80	5.19 ± 0.73^ns^
Kidney	21.6 ± 3.84	24.1 ± 3.54^ns^
Muscle	0.40 ± 0.06	0.53 ± 0.07^ns^
Tumor	2.89 ± 0.84	2.17 ± 0.84^a^
Tumor MVD	4.65 ± 1.15	4.09 ± 1.38^b^

Tissue radioactivity was assessed 3 h after injection of 0.74 MBq of the tracer and is expressed as % ID/g (mean ± SD). The corresponding tumor MVD was presented as a percentage of the CD31-positive area

^ns^ *P* > 0.05; ^a^ *P* = 0.085; ^b^ *P* = 0.36 versus the vehicle-treated group

### PET imaging of the antiangiogenic effect of TSU-68

**Fig. 4:**
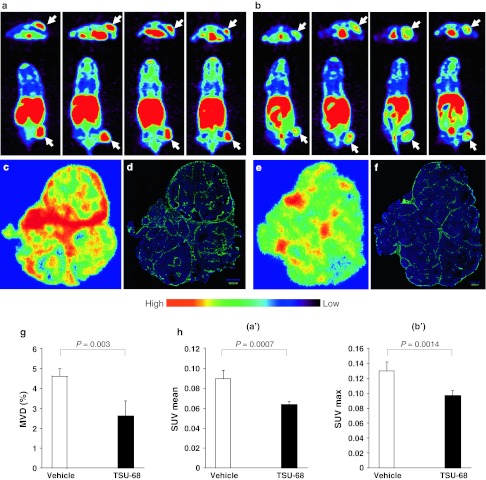
Transverse and coronal PET images of s.c. HuH-7 tumor-bearing mice at 3 h after i.v. injection of ^64^Cu-cyclam-RAFT-c(-RGDfK-)_4_ (11.1 MBq) on the day after daily i.p. injections of (**a**) vehicle alone (50 μl of DMSO) or (**b**) TSU-68 (75 mg kg^−1^ d^−1^ in 50 μl of DMSO) for 14 days (n = 4 mice for each group). The arrows indicate the tumor location. Representative autoradiographic examination (**c**, **e**) and CD31 immunofluorescence staining (**d**, **f**) with the same whole-tumor sections from (**c**, **d**) vehicle- and (**e**, **f**) TSU-68-treated tumors excised after PET imaging. **g** MVD, **ha′** SUV_mean_, and **hb′** SUV_max_ were compared between TSU-68- and vehicle-treated tumors. All data presented in **a**–**h** are from the same set of experimental groups

Transverse and coronal PET images from the vehicle and TSU-68-treated mice (n = 4 for each group) acquired at 3 h p.i. of ^64^Cu-cyclam-RAFT-c(-RGDfK-)_4_ are shown in Fig. 4a and b, respectively. Visually, the radioactivity was obviously lower in the tumors from the TSU-68-treated mice than in those from the controls. Further, while the radioactivity accumulated in the control tumors was homogeneous, the radioactivity signals in the TSU-68-treated tumors were heterogeneous: higher activity was observed in the tumor rim than in the center, as shown in both the transverse and coronal PET images.

Autoradiographic examination and CD31 immunofluorescence staining of the tumor microvessels with the same whole-tumor sections revealed well-matched intratumoral localization between ^64^Cu-cyclam-RAFT-c(-RGDfK-)_4_ and microvessels in both the vehicle (Fig. 4c, d) and TSU-68-treated tumors (Fig. 4e, f). As compared to the dense network of microvessels observed in vehicle-treated tumors, the microvessels of TSU-68-treated tumors appeared sparse and lacking interconnection.

As seen in Fig. 4g, the MVD was significantly lower in the TSU-68-treated tumors that in the control tumors (2.63 ± 0.71 % vs. 4.61 ± 0.37 %; *P* = 0.003), by 43 %. Quantitative image analysis showed that both the SUV_mean_ (Fig. 4h, a′) and SUV_max_ (Fig. 4h, b′) were significantly lower in the TSU-68-treated tumors than in the control tumors (SUV_mean_, 0.065 ± 0.002 vs. 0.091 ± 0.007, *P* = 0.0070; SUV_max_, 0.105 ± 0.097 vs. 0.137 ± 0.012, *P* = 0.0014), by 29 and 23 %, respectively. No significant difference in SUV_min_ was observed between the groups (data not shown). When the tumor SUV and corresponding MVD were analyzed, a positive and significant correlation was observed between the SUV_mean_ and MVD and between the SUV_max_ and MVD, though the correlation coefficient was higher for SUV_mean_ (r = 0.829, *P* = 0.011) than for SUV_max_ (r = 0.776, *P* = 0.024) (Supplemental Fig. 2).

When serial tumor sections were stained for CD31 and CD105 and visualized using the same fluorescent dye-conjugated secondary antibody, no detectable difference in the localization pattern was found between CD31 and CD105 (Supplemental Fig. 3). Immunofluorescence double staining of CD31 and CD61 revealed that CD61 predominantly colocalized with CD31, but not all CD31-positive endothelial cells colocalized with CD61, indicating that murine β_3_ integrin is expressed mainly on activated endothelial cells (Supplemental Fig. 4).

Next, autoradiographic examination of kidney sections from control and TSU-68-treated mice showed that the radioactivity signal was predominantly localized in the cortex for both groups, and TSU-68 treatment resulted in reduced activity retention but seemed to have no effect on the distribution pattern (Supplemental Fig. 5). Immunofluorescence double staining of CD31 and CD61 on the same kidney sections did not reveal an obvious difference in the CD31 staining between the TSU-68- and vehicle-treated mice, and the CD61 staining was negligible in both groups (Supplemental Fig. 5).

## Discussion

α_V_β_3_ Integrin is strongly expressed on activated endothelial cells during angiogenesis and on some types of tumor cells [27]. Therefore, the α_V_β_3_ integrin expression levels in a tumor may predominantly be associated with its vasculature or with the vasculature and α_V_β_3_-positive tumor cells. Thus far, an increasing number of radiolabeled cRGD-containing peptides have been developed using various strategies for peptide modification and radiolabeling techniques for imaging α_V_β_3_ integrin [14]. Since most studies used α_V_β_3_-positive tumor cells to establish the tumor model, and tumor vasculature represents about 5 % of the volume, the effectiveness of the RGD-based PET probe to detect angiogenesis requires greater validation. In the present study, we selected tumor xenografts derived from the α_V_β_3_-negative tumor cell line HuH-7 to eliminate interference from α_V_β_3_ integrin expressed on the tumor cells themselves. Another reason we selected this model is that it showed strong angiogenesis when subcutaneously transplanted into nude mice. RAFT-c(-RGDfK-)_4_ with or without radio- or fluorescent dye labeling has been shown to have higher α_V_β_3_ integrin-binding specificity and enhanced binding activity than its monomeric analog in both in vitro and in vivo studies using different cell lines and murine tumor xenografts [22–25, 28–30]. Here, we prove that ^64^Cu-cyclam-RAFT-c(-RGDfK-)_4_ PET enabled visualization of tumor angiogenesis by targeting α_V_β_3_ integrin. The imaging quality was good, and this finding was supported by a biodistribution assay, which showed high tumor-to-blood and tumor-to-muscle ratios (31.6 and 6.7, respectively, at 3 h p.i.). Our results are in agreement with those of Haubner et al. [31], showing that ^18^F-galacto-RGD PET using the α_V_β_3_-negative A431 tumor model can enable the visualization of α_V_β_3_ integrin expression resulting exclusively from tumor vasculature in mice.

As mentioned earlier, a few studies have reported the use of radiolabeled cRGD-containing peptides to monitor the response of tumors to angiogenesis inhibitors in animal models [10, 11, 20, 21]. Battle et al. [21] showed the inhibitory effect of sunitinib, a Food and Drug Administration (FDA) approved multi-targeted receptor tyrosine kinase (RTK) inhibitor, on the uptake of ^18^F-fluciclatide (formerly known as ^18^F-AH111585) by α_V_β_3_-positive U87MG tumors as revealed by repeated PET imaging during the 2-weeks treatment period; they also found a reduction in the tumor MVD at the end of the therapy. Compared with the monomeric peptide tracer of ^18^F-fluciclatide, the dimeric peptide FPPRGD2 labeled with ^18^F showed higher α_V_β_3_-specific accumulation and better pharmacokinetics [32]. Chen’s group reported the use of ^18^F-FPPRGD2 for PET imaging of the tumor response to antiangiogenic therapy with ZD4190 [11] or Abraxane [10] in mice bearing α_V_β_3_-positive MDA-MB-435 breast tumors. ZD4190, an RTK inhibitor of vascular endothelial growth factor (VEGF) receptor, appeared to exert an acute effect on α_V_β_3_ integrin expression by downregulating the levels on both tumor cells and the endothelium as early as 2 h after the first drug administration, and this finding corresponded with reduced tumor ^18^F-FPPRGD2 uptake. Abraxane, an FDA-approved nanoparticle albumin-bound paclitaxel, showed antiangiogenic activity at a low dose (25 mg kg^−1^). Significantly decreased tumor tracer uptake was found as early as 3 days after a single dose of Abraxane administration, correlating with a significant reduction in the β_3_ integrin expression on the endothelium and no change in α_V_β_3_ integrin expression on the tumor cells themselves. Collectively, the results demonstrate that PET with an RGD peptide tracer is promising for the assessment of antiangiogenic effects. However, they also indicate that some antiangiogenic drugs such as ZD4190 may affect tumor cells expressing α_V_β_3_ integrin, making it difficult to directly correlate the reduction in tumor tracer uptake with the reduction in tumor MVD.

In our previous study, we found a strong and positive correlation between tumor uptake levels of ^64^Cu-cyclam-RAFT-c(-RGDfK-)_4_ and the corresponding α_V_β_3_ integrin expression levels quantified by SDS-PAGE/autoradiography [24]. Here, we used this tracer to monitor the tumor response to the antiangiogenic drug TSU-68, a novel and selective RTK inhibitor of the angiogenic receptors for VEGF, platelet-derived growth factor, and fibroblast growth factor. Preclinical studies have shown that oral or i.p. administration of TSU-68 resulted in significant growth inhibition of tumor xenografts of diverse origins [33]. TSU-68 clinical trials are ongoing for patients with different advanced cancers such as hepatocellular carcinoma, gastrointestinal cancer, breast cancer, and non-small cell lung cancer. In an experimental study of C6 glioma xenografts in mice, daily i.p. administration of 75 mg kg^−1^ d^−1^ TSU-68 in 50 μl of DMSO resulted in a significant reduction in the tumor MVD after >10 days of therapy [33]. A similar TSU-68 dosage was used in the present study, and it significantly delayed tumor growth and reduced tumor MVD in mice bearing HuH-7 xenografts after 14 days of therapy. The results obtained from the same set of experiments showed that the reduction in tumor MVD induced by TSU-68 treatment was accompanied by a reduction in the tumor SUV as quantified by PET imaging, and these results were further supported by those of the biodistribution assay. More importantly, a positive and significant correlation was found between the tumor MVD and the corresponding SUV (either the mean or maximum value) or % ID/g of tumor uptake evaluated using the biodistribution assay. The intratumoral colocalization of the tracer and vascular network distribution and the colocalization of CD31 and murine β_3_ integrin support these results, which strongly demonstrate that the antiangiogenic effects of TSU-68 can be monitored by quantitative ^64^Cu-cyclam-RAFT-c(-RGDfK-)_4_ PET imaging. In clinical studies, the treatment cycle for TSU-68 is usually daily administration for 28 days, and some patients even took the drug for >6 months [34, 35]. In the present small animal imaging study, ^64^Cu-cyclam-RAFT-c(-RGDfK-)_4_ PET showed significant changes in tumor tracer uptake after 2 weeks of drug administration. Although it is difficult to predict how early the antiangiogenic effect can be imaged, we believe that early detection is possible since the decrease in the tumor growth rate was observed as early as day 3 (after 2 injections of the drug) and because the decreasing tendency of tumor tracer uptake was observed after only 1 day of drug injection. In addition to its antiangiogenic effect on endothelial cells, the direct effect of TSU-68 on tumor cell proliferation must be recognized. Periodic imaging during the course of treatment would be valuable to determine the time point at which the response can be monitored, and we intend to conduct such a study in the future. The value of RGD PET for assessing and evaluating antiangiogenic treatment is that it is not only noninvasive and quantitative but also can evaluate the activation status of the tumor vasculature by targeting α_V_β_3_ integrin expressed on proliferating endothelial cells. Moreover, it helps determine whether an antiangiogenic drug is effective in individuals by enabling the assessment of alterations in tracer uptake not only in terms of the intensity but also the distribution.

Two weeks of TSU-68 administration did not cause a significant reduction in body weight, and no other side effects were noted. However, a biodistribution assay for ^64^Cu-cyclam-RAFT-c(-RGDfK-)_4_ showed that the radioactivity accumulated in most of the normal organs of the TSU-68-treated mice was significantly reduced. One possible explanation for this is that TSU-68 indirectly affects the expression pattern and/or activation state of integrins despite their low levels in normal tissues. Autoradiographic examination and immunostaining of endothelium/murine β_3_ integrin in kidney sections indicated that the reduced renal radioactivity was not due to the antiangiogenic effect of TSU-68, supporting this explanation.

The maximum radioactivity accumulation of ^64^Cu-cyclam-RAFT-c(-RGDfK-)_4_ was found in the kidneys. The negligible levels of β_3_ integrin expression in the kidney together with our previous finding [24] that ^64^Cu-cyclam-RAFT-c(-RGDfK-)_4_ renal radioactivity could not be blocked by excessive amounts of “cold” α_V_β_3_-specific RGD peptides strongly supported the conclusion that the kidney acts as the main excretory organ for ^64^Cu-cyclam-RAFT-c(-RGDfK-)_4_. Renal radioactivity was observed predominantly in the cortex, the same site as that reported for other radiolabeled peptides [36, 37], such as ^111^In-DOTA,Tyr^3^-octreotate, whose radioactivity retention was significantly reduced by the coadministration of Gelofusine, a gelatin-based plasma expander [36]. It would be worthwhile to determine whether Gelofusine is also effective in the case of ^64^Cu-cyclam-RAFT-c(-RGDfK-)_4_, since the decrease in renal radioactivity may contribute to improving imaging quality, especially for disorders of the abdomen. Gelofusine may also reduce the risk of nephrotoxicity when ^64^Cu (^67^Cu)-cyclam-RAFT-c(-RGDfK-)_4_ is used for internal radiotherapy.

^18^F-labeled RGD peptides including galacto-RGD [31], fluciclatide [38], and FPPRGD2 [39] have been used to treat cancer patients, and the FDA has approved the use of ^18^F-FPPRGD2 PET/CT imaging for evaluation of antiangiogenic therapy in solid tumors. However, the procedures for preparing such tracers are rather complex and time consuming, which limits their widespread clinical use [40]. We previously reported ^64^Cu-radiolabeling of cyclam-RAFT-c(-RGDfK-)_4_ [24] and then made further improvements to achieve a specific radioactivity as high as ~37 MBq/nmol with a labeling efficiency >99 %, making purification unnecessary. Further, metabolic analysis of this agent in mice showed its high in vivo stability (unpublished data). Moreover, the labeling procedure is straightforward: the mixture of peptide and ^64^Cu only needs to be incubated at 37 °C for less than 60 min. The ease of preparation, high radiochemical yield, and high metabolic stability make ^64^Cu-cyclam-RAFT-c(-RGDfK-)_4_ a promising PET probe for α_V_β_3_ integrin imaging.

In conclusion, we used murine xenografts from an α_V_β_3_-negative tumor cell line and showed that ^64^Cu-cyclam-RAFT-c(-RGDfK-)_4_ PET enables the clear visualization of tumor angiogenesis and helps monitor the effectiveness of antiangiogenic therapy. In future studies, we intend to determine whether this strategy is effective for tumors in which α_V_β_3_ is expressed on both tumor cells and the neovasculature by using longitudinal PET imaging to detect not only changes in tracer uptake but also changes in the tracer distribution pattern. With advances in technology, the spatial resolution of PET is expected to improve greatly to enable clear visualization of intratumoral tracer distribution. Because of the good imaging quality and easy preparation, ^64^Cu-cyclam-RAFT-c(-RGDfK-)_4_ is a promising PET tracer for tumor angiogenesis imaging. Further, it may also be applicable for monitoring angiogenic therapy in other angiogenesis-associated disorders such as ischemia, atherosclerosis, and myocardial infarction.

## Electronic supplementary material

Below is the link to the electronic supplementary material.

Supplementary material 1 (PDF 166 kb)
