# Supplementary material for: Positron emission tomography imaging of tumor angiogenesis and monitoring of antiangiogenic efficacy using the novel tetrameric peptide probe 64Cu-cyclam-RAFT-c(-RGDfK-)4
Source: Angiogenesis. 2012 May 29;15(4):569–80. doi: 10.1007/s10456-012-9281-1 (PMC3496517; doi:10.1007/s10456-012-9281-1)

Supplemental Figure 1

Correlation between tumor ^{64}Cu-cyclam-RAFT-c(-RGDfK-)$_4$ uptake expressed as %ID/g (mean ± SD) and corresponding HuH-7 tumor MVD (%). %ID/g was measured by biodistribution assay

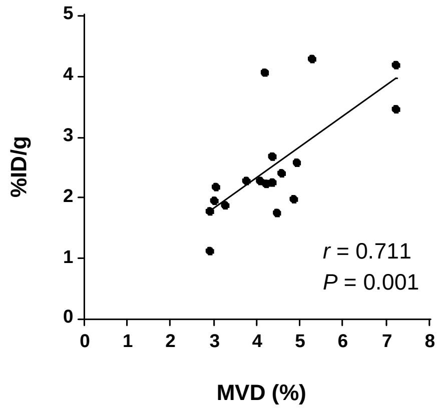

Supplemental Figure 2

Correlation between tumor ^{64}Cu-cyclam-RAFT-c(-RGDfK-)$_4$ uptake expressed as SUV (mean or max) and corresponding HuH-7 tumor MVD (%). SUV was measured by quantative PET imaging

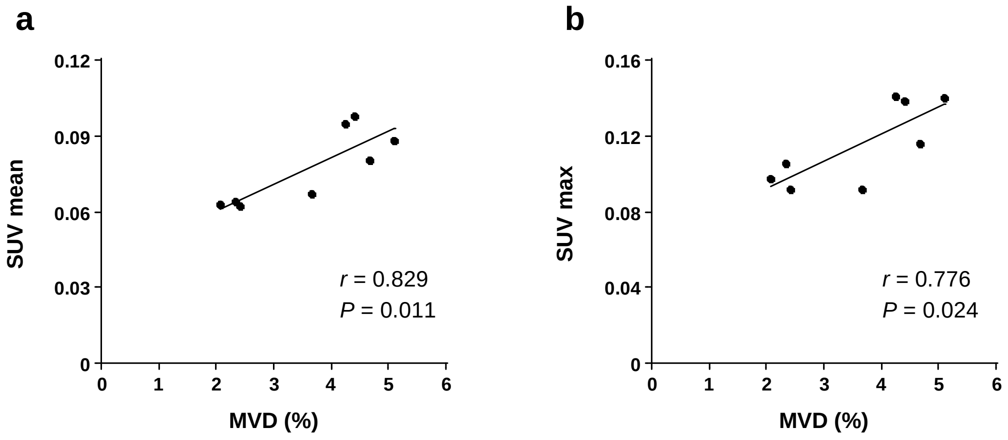

Supplemental Figure 3

Immunofluorescence staining of CD31 and CD105
of serial HuH-7 tumor sections

CD31

CD105

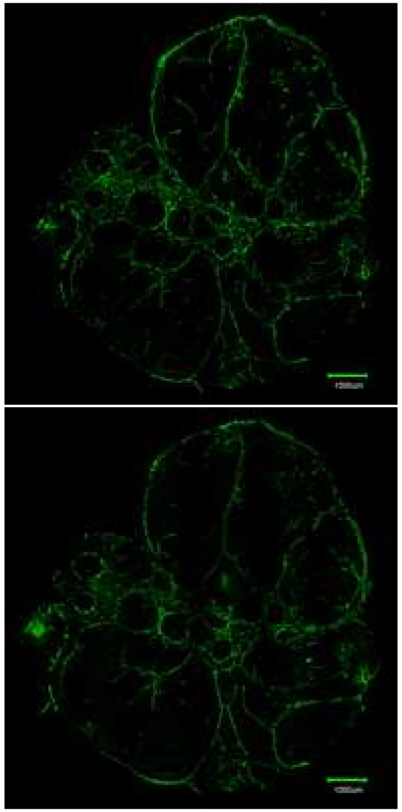

Supplemental Figure 4

Double immunofluorescence staining of CD31 and CD61
of HuH-7 tumor section, with nuclei stained with DAPI

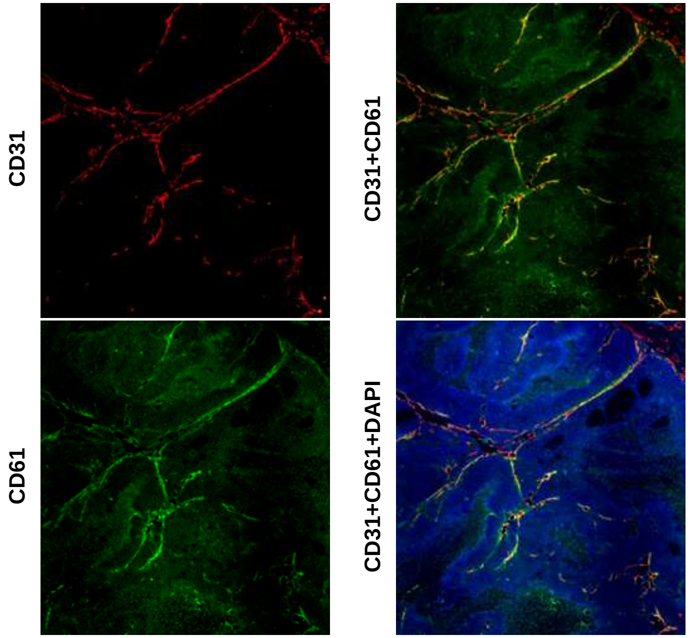

Supplemental Figure 5

Autoradiographic examination and double immunofluorescence staining of CD31 and CD61
with the same kidney sections from mice treated with vehicle or TSU-68, with nuclei stained with DAPI

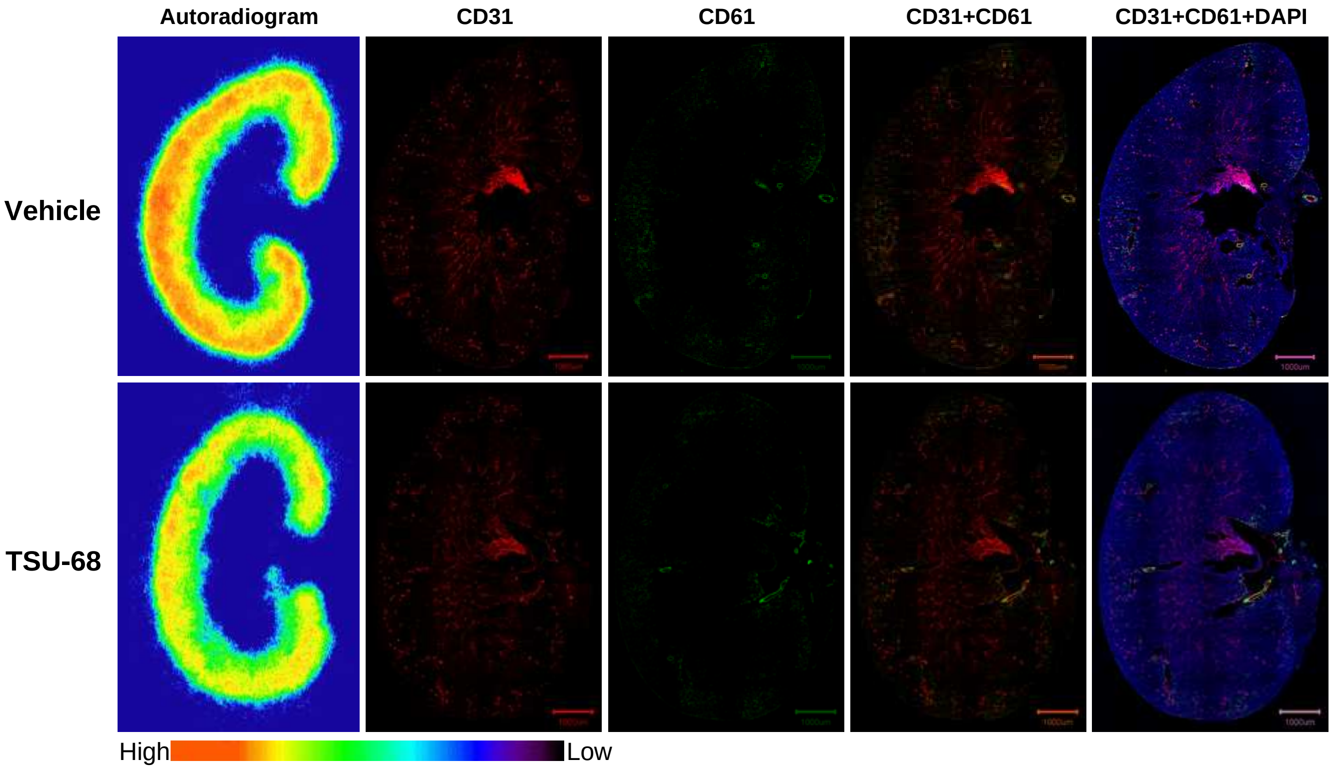

Supplement: Supplementary file 1 — Supplementary material 1 (PDF 166 kb) [file 10456_2012_9281_MOESM1_ESM.pdf]
